# Transcriptomic response for revealing the molecular mechanism of oat flowering under different photoperiods

**DOI:** 10.3389/fpls.2023.1279107

**Published:** 2023-10-31

**Authors:** Man Zhang, Yuan Jiang, Haixiao Dong, Xiaohui Shan, Juan Tian, Moke Sun, Feiyue Ma, Changzhong Ren, Yaping Yuan

**Affiliations:** ^1^ Jilin Engineering Research Center for Crop Biotechnology Breeding, College of Plant Science, Jilin University, Changchun, China; ^2^ Key Laboratory of Biotechnology of Jinlin Province, Baicheng Academy of Agricultural Science, Baicheng, China

**Keywords:** oat, photoperiod, transcriptome, flowering, molecular mechanism

## Abstract

Proper flowering is essential for the reproduction of all kinds of plants. Oat is an important cereal and forage crop; however, its cultivation is limited because it is a long-day plant. The molecular mechanism by which oats respond to different photoperiods is still unclear. In this study, oat plants were treated under long-day and short-day photoperiods for 10 days, 15 days, 20 days, 25 days, 30 days, 40 days and 50 days, respectively. Under the long-day treatment, oats entered the reproductive stage, while oats remained vegetative under the short-day treatment. Forty-two samples were subjected to RNA-Seq to compare the gene expression patterns of oat under long- and short-day photoperiods. A total of 634-5,974 differentially expressed genes (DEGs) were identified for each time point, while the floral organ primordium differentiation stage showed the largest number of DEGs, and the spikelet differentiation stage showed the smallest number. Gene Ontology (GO) analysis showed that the plant hormone signaling transduction and hormone metabolism processes significantly changed in the photoperiod regulation of flowering time in oat. Moreover, Kyoto Encyclopedia of Genes and Genomes (KEGG) and Mapman analysis revealed that the DEGs were mainly concentrated in the circadian rhythm, protein antenna pathways and sucrose metabolism process. Additionally, transcription factors (TFs) involved in various flowering pathways were explored. Combining all this information, we established a molecular model of oat flowering induced by a long-day photoperiod. Taken together, the long-day photoperiod has a large effect at both the morphological and transcriptomic levels, and these responses ultimately promote flowering in oat. Our findings expand the understanding of oat as a long-day plant, and the explored genes could be used in molecular breeding to help break its cultivation limitations in the future.

## Introduction

As one of the most important small grain crops, oat (*Avena sativa* L.) provides extremely high nutritional value to people and plays an important role in improving people’s diet structure (Kamal et al., 2022; Qin et al., 2023). Oat is generally classified as a long-day plant since most cultivars cannot flower to produce seeds under a short-day photoperiod (day length shorter than 12 hours) (Trevaskis et al., 2022). Flowering is a crucial event in the plant life cycle that determines grain yield and quality and is influenced by various endogenous and exogenous factors (Liu et al., 2020). Previous studies have shown that many plants must undergo a suitable photoperiod for a certain amount of time before they can flower. Short-day plants flower under shortened daylength. Conversely, long-day plants flower when the daylength is extended (Garner and Allard, 1920). The change in plant sensing day length plays an important biological function, reflecting its adaptation to environmental changes. Therefore, photoperiod is one of the most important environmental factors that affect flowering time in plant species (Zheng et al., 2019).

In *Arabidopsis*, the photoperiod master regulator gene *CONSTANS* (*CO*) plays a connecting role between the circadian clock and flowering time genes (Putterill et al., 1995). The circadian clock controls the transcription level of CO through circadian rhythm-regulated components, such as clock-controlled blue light photoreceptor FLAVINBINDING, KELCH REPEAT, F-BOX1 (FKF1) and clock-controlled CYCLING DOF FACTOR (CDF) transcription factors (Fornara et al., 2009; Hayama et al., 2017). Under long-day conditions, CDF1 directly binds the promoter of *CO* to inhibit its transcription in the morning, while the FKF1-GI complex activates *CO* transcription by binding to and initiating the degradation of CDFs in the afternoon (Andrés and Coupland, 2012; Song et al., 2015). Under short-day conditions, insufficient FKF1 is generated; therefore, *CO* transcription is always inhibited (Valverde et al., 2004). Moreover, several clock components such as PSEUDO RESPONSE REGULATORs (PRRs) mediate the stabilization of CO by interacting with CO, and this stabilization can increase the capacity of CO to bind to the promoter of FLOWERING LOCUS T (FT), leading to enhanced FT transcription and early flowering under LDs and SDs (Hayama et al., 2017). Similar flowering regulatory mechanisms have been discovered in rice. For rice, a short-day plant, *HD1* and *HD3a* are homologs of *AtCO* and *AtFT*, respectively, and the GI-CO-FT signaling pathway is also evolutionarily conserved in rice (Yano et al., 2000; Kojima et al., 2002; Zhou et al., 2021). Previous studies have reported that the photoperiod response is mainly mediated by photoperiod-H1 (Ppd-H1) in barley, similar to *Arabidopsis* PRR7, which positively regulates the *FT* gene under a long-day photoperiod (Turner et al., 2005; Digel et al., 2015). In wheat, three orthologs of barley Ppd-H1 were identified and shown to have the greatest contribution to flowering regulation (Beales et al., 2007; Shimada et al., 2009). For example, the *PPD1* genes and CO1 are able to respond to the photoperiod in the absence of each other, indicating that *PPD1* genes have a special role in the photoperiod pathway of wheat (Pearce et al., 2017; Shaw et al., 2020). In summary, the research on these genes and regulatory flowering pathways from different plants was not exactly the same, and the above studies contribute to systematically studying the photoperiodic responses in oat.

In addition to the photoperiodic pathway, hormone metabolism, hormone signaling transduction and carbohydrate metabolism also play important regulatory roles in flowering regulation (Wahl et al., 2013; Ionescu et al., 2017; Freytes et al., 2021). For example, the biological function of gibberellin (GA) in flowering time control mainly depends on the growth repressor DELLA proteins (Bao et al., 2020). High CK levels cause a dwarf phenotype with early flowering time (Gawarecka and Ahn, 2021). In contrast, abscisic acid (ABA) treatment greatly delays flowering (Wang et al., 2013). Mutants of the ABSCISIC ACID-INSENSITIVE 3 (ABI3), ABI4 and ABI5 genes showed early flowering (Zhang et al., 2005; Wang et al., 2013; Shu et al., 2016). Additionally, carbohydrate substances have a significant regulatory effect on the flowering process. Exogenous sucrose can directly increase the expression of *FT* genes and promote the flowering of chrysanthemum (*Chrysanthemum morifolium*) under long-day and short-day conditions (Sun et al., 2017). The mutant of the rice invertase gene Inv1 showed a late-flowering phenotype (Jia et al., 2008). Above all, hormone regulation and sugar metabolism are the essential parts of flowering time control.

For oat, several strategies have been applied to identify several QTLs associated with flowering time using restriction fragment length polymorphism (RFLP) and single nucleotide polymorphism (SNP). Eight loci for days to heading on linkage groups 3, 7, 8, 11, 12, 17 and 24 were detected throughout the oat genome in the Kanota×Ogle population (Holland et al., 1997). Moreover, three major flowering-time QTLs (in linkage groups OT8, OT31 and OT32) were detected using the Ogle×TAM O-301 population under four combinations of photoperiod and vernalization treatments with RFLP (Holland et al., 2002). With the single-nucleotide polymorphism (SNP) strategy, numerous regions of the genome in the linkage groups Mrg02, Mrg12, Mrg13, and Mrg24 were associated with heading date within location years. Using the above oat map, three major QTLs controlling heading date were identified in the populations (Esvelt Klos et al., 2016; Zimmer et al., 2018). A recent study showed that combined transcriptome sequencing was performed using the developing leaves and main shoot apices (MSAs) of photoperiod-sensitive and photoperiod-insensitive varieties under long-daylength conditions (12 h light/12 h dark), and the results revealed that the photoperiod and CK pathways could regulate the photoinsensitivity of oat (An et al., 2020). However, no efforts have been made to develop a more extensive understanding of the molecular mechanism that controls flowering time in photoperiod-sensitive oats.

As an important cereal and forage crop, the cultivation of oat is limited by its feature of being a long-day plant (Marshall et al., 1992; Arjona et al., 2020). Therefore, exploring the important flowering genes and understanding the flowering mechanism of oat under different photoperiods is of great significance and would help overcome these limitations. In this study, RNA sequencing (RNA-seq) was conducted on oat leaves at different times under long-day and short-day photoperiods. The differentially expressed genes (DEGs) in seven comparison groups annotated by the Gene Ontology (GO) and Kyoto Encyclopedia of Genomes and Genomes (KEGG) databases showed that the regulation of many flowering pathways, including photoperiod, plant hormone, circadian clock and sugar metabolism were critical for flowering time control in oat. This study could facilitate a deep understanding of the molecular mechanism underlying the photoperiod response of oat and provide an important reference for the molecular breeding of oat.

## Materials and methods

### Plant materials, treatments, and sample collection

Baiyan 2, a photoperiod-sensitive variety, was used in the photoperiod experiment. Seeds were sown in plastic pots in the greenhouse. The same water management strategy was used for all plants. All plants were kept in the greenhouse for two weeks under short-day conditions (10 h light at 24°C and 14 h dark at 20°C) with 8000 lux light intensity. When the plants were at the first leaf fully expanded stage, half of the plants were shifted to long-day conditions until spikelets fully emerged. The long-day photoperiod treatment was initiated at 8:00 am and ended at 10:00 pm, and the short-day treatment was initiated at 8:00 am and ended at 6:00 pm. The uppermost unfolded leaves were sampled in three replicates at 10:00 am under long-day photoperiod conditions, and each replicate was pooled from 3 plants. The SD samples used as the control were also collected at the same time (**Supplementary Table 1**). Then, the samples were immediately frozen in liquid nitrogen and stored at −80°C.

### Phenotypes of the shoot apical meristem by scanning electron microscopy

The shoot apical meristems (SAM) were removed manually, and then the hand-dissected apices were fixed in 5% FAA (5% formaldehyde, 50% ethanol and 5% acetic acid). The phenotype of each SAM was observed using a scanning electron microscope XL-30FE-ESEM FEG (FEI Company, America).

### RNA extraction and sequencing

Total RNA was extracted using an Ultrapure RNA Kit from Beijing ComWin Biotech Co., Ltd. (China) following the manufacturer’s protocol. RNA quality and quantity were assessed by a NanoDrop 2000 UV Spectrophotometer (Thermo Fisher Scientific) and an Agient2100 Bioanalyzer (Agilent,Santa Clara, CA, USA). Forty-two samples were subjected to RNA-Seq using an Illumina Noveseq6000 platform of Biomarker Technologies Co., Ltd. (Beijing,China). The raw data were uploaded to the NCBI Sequence Read Archive (http://www.ncbi.nlm.nih.gov/) with the accession number PRJNA997076. The clean reads were mapped to the reference genome (https://www.ncbi.nlm.nih.gov/datasets/genome/GCA_916181665.1/) using HISAT2 (Kim et al., 2019).

### Mining and functional investigation of DEGs

The expression levels of genes were determined by calculating fragments per kilobase of transcript per million fragments mapped (FPKM) using StringTie software. The DEGs were identified by DESeq2. The threshold was set to: a false discovery rate (FDR) ≤0.05 and a |fold change|≥1.5. Venn graphs and heatmaps were drawn by TBtools v1.132 (Chen et al., 2020).

Gene Ontology (GO) enrichment analysis was performed on the BMKCloud platform (www.biocloud.net). Significant enrichment of GO terms and KEGG pathways was set at a q-value<0.05. The DEGs were annotated with Mercator version 3.6 online and evaluated with MapMan functional annotation (version 3.6.0) (Thimm et al., 2004).

### Quantitative real-time PCR

Total RNA was reverse transcribed to synthetize first-strand cDNA using the UEIris II RT-PCR System for First-Strand cDNA Synthesis with dsDNase (US Everbright). qRT-PCR was performed using the PCRmax Eco 48 real-time PCR machine (PCRMax, Staffordshire, UK). Primers for qRT-PCR are listed in **Supplementary Table 2**. Glyceraldehyde-3-phosphate dehydrogenase (GAPDH) (AK251456) was used as the endogenous reference gene (Jarosová and Kundu, 2010).

The qRT-PCR system program followed the instructions of the 2×SYBR Green qPCR Master Mix (Bimake). The relative expression of the genes was calculated using the comparative 2-ΔΔCT method (Livak and Schmittgen, 2001). Three technical replicates were performed for each gene.

## Results

### Morphological changes in the SAM under a long-day photoperiod

**Figure 1:**
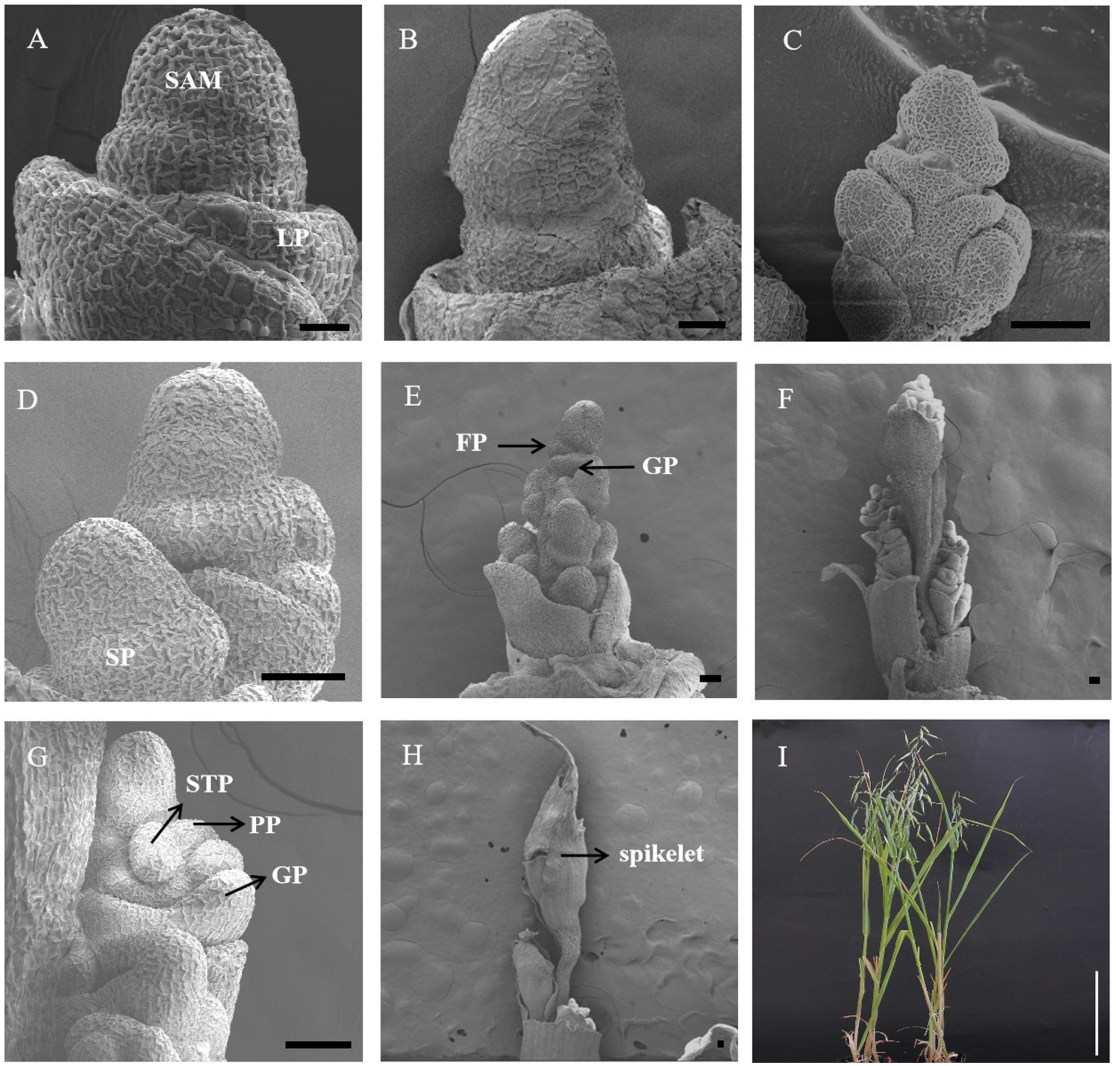
Scanning electronic micrographs of SAM under short-day conditions **(A)** and different long-day inducement periods **(B–H)**. **(A)** The vegetative stage of the apical meristem (10SD-50SD). **(B)** The enlargement stage of the apical dome (10LD). **(C)** Branch differentiation stage (15LD). **(D)** Spikelet differentiation stage (20LD). **(E)** Florets differentiation stage (25LD). **(F, G)** Floral organ primordium differentiation stage (30LD). **(H)** The booting stage (40LD). **(I)** The heading date stage. LP, leaf primordium; SP, spikelet primordium; FP, floret primordium; GP, glume primordium; STP, stamen primordial; PP, pistil primordial; GP, glume primordium. Black bar=50 μm. White Bar=20 cm.

After long-day photoperiod induction, oat SAMs at different developmental stages were collected and observed by scanning electron microscopy. As shown in **Figure 1** and **Supplementary Figure 1**, the phenotypes of the SAM changed and progressed to the reproductive stage, including the elongation stage, branch differentiation stage, spikelet differentiation stage, floret differentitation stage, floral organ primordium differentiation stage, tetrad stage (booting stage), and heading date stage after 10/15/20/25/30/40/50 days of a long-day photoperiod, while the SAM was still in the vegetative stage under SD conditions. At the 30 long-day photoperiod, the meristem of oat was further differentiated, forming the glume primordium, pistil primordium and stamen primordium (**Figure 1G**). These results indicate that the spike architecture was shaped by environmental conditions and that floral induction was significantly promoted under the long-day photoperiod treatment.

### Identification of differentially expressed genes under the photoperiod induction

The leaves of the photoperiod-sensitive variety Baiyan 2 were sampled at 10 days, 15 days, 20 days, 25 days, 30 days, 40 days and 50 days of long-day and short-day photoperiods. Forty-two samples were subjected to RNA-Seq using the Illumina NoveSeq6000 sequencing platform. After removing the low-quality reads, a total of 902,814,239 clean reads with 270,428,539,220 nucleotides were obtained. The GC content of each sample was more than 53.32%, and the Q30 percentage ranged from 92.39% to 95.61%. Then, the high-quality reads from each sample were mapped to the oat reference genome, and the unique mapped ratios ranged from 82.31% to 86.51% (**Supplementary Table 3**), indicating that the OT3098 v2 hexaploid oat genome fulfilled the demand for information analysis and that further data analysis was reliable. Pearson r2 correlation values for three replicates from seven groups varied from 0.80 to 0.95 (**Supplementary Table 4**), indicating the acceptable reproducibility of the raw data.

**Figure 2:**
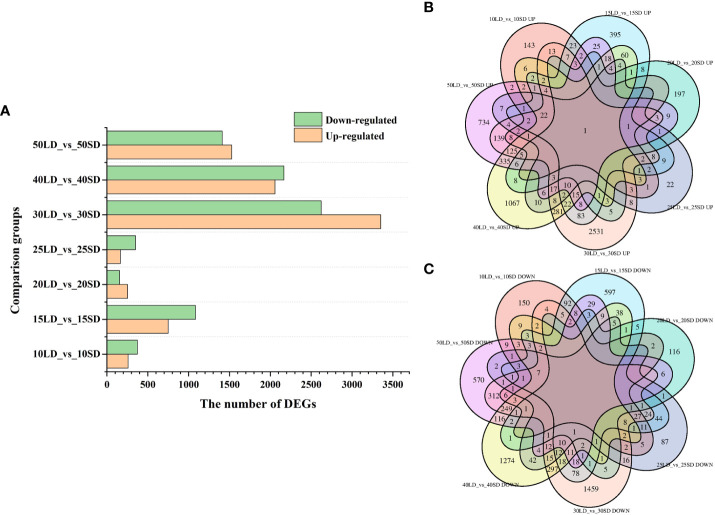
Differentially expressed gene (DEG) analysis under different photoperiods. **(A)** Number of upregulated or downregulated DEGs in seven pairwise comparison periods. **(B)** Venn diagram displaying only one common DEG that was upregulated in seven comparisons. **(C)** Venn diagram displaying no common DEG that was downregulated in seven comparisons.

With the standards of |fold changes|≥1.5 and FDR ≤ 0.05, a total of 12,054 DEGs were identified between the long-day and short-day conditions and the list of DEGs in the seven comparison groups (634 in 10LD_vs_10SD, 1834 in 15LD_vs_15SD, 407 in 20LD_vs_20SD, 515 in 25LD_vs_25SD, 5,974 in 30LD_vs_30SD, 4,224 in 40LD_vs_40SD and 2,938 in 50LD_vs_50SD) are shown in **Supplementary Table 5**. Among the seven groups, the highest number of DEGs was identified in 30LD_vs_30SD, with 3,352 and 2,622 up- and downregulated genes, respectively (**Figure 2A**). The lowest number of DEGs was observed in 20LD_vs_20SD, with only 253 upregulated and 154 downregulated genes (**Figure 2A**). The Venn diagram showed that only one upregulated gene was shared among the seven comparison groups (**Figure 2B**), while no downregulated gene existed in all comparisons (**Figure 2C**).

### Transcriptomic responses upon flowering under different photoperiods

Moreover, to investigate the mechanisms of flowering time at the molecular level, we performed gene ontology analysis on the DEGs by using the BMKCloud online tool and mainly analyzed the BP category terms to detect the essential function of these DEGs in the response to different photoperiod periods. To reduce redundancy, we only retained child GO terms in this study and removed the parent terms with hierarchical relationships.

A total of 32 (11 for up-DEGs and 21 for down-DEGs), 103 (32 for up-DEGs and 71 for down-DEGs), 15 (10 for up-DEGs and 5 for down-DEGs), 27 (10 for up-DEGs and 17 for down-DEGs), 209 (73 for up-DEGs and 136 for down-DEGs), 150 (67 for up-DEGs and 83 for down-DEGs), and 99 (46 for up-DEGs and 53 for down-DEGs) related BP terms were enriched after growth in a long-day photoperiod for 10/15/20/25/30/40/50 days in oat (**Supplementary Table 6**). Generally, we found that fewer BP terms were enriched in the early flowering stage except at 15 days, but more BP terms were enriched in the last three periods, indicating that oat has more complex regulatory mechanisms in the later flowering stage.

For the GO terms at 10 days, ‘polysaccharide catabolic process’ was the most significant term enriched by the up-DEGs, and ‘photosynthesis, light harvesting in photosystem I’ was the most significant term enriched by the down-DEGs. For the GO terms at 15 days, ‘polysaccharide catabolic process’ and ‘cellular response to phosphate starvation’ were the most significant terms enriched by the up-DEGs, and ‘protein-chromophore linkage’ and ‘photosynthesis, light harvesting in photosystem I’ were the most significant terms enriched by the down-DEGs. For the GO terms at 20 days, ‘collagen catabolic process’ was the most significant term enriched by the up-DEGs, and ‘positive regulation of rRNA processing’ was the most significant term enriched by the down-DEGs. For the GO terms at 25 days, ‘intracellular sequestering of iron ion’ was the most significant term enriched by the up-DEGs. For the GO terms at 30 days, ‘ribosomal large subunit assembly’ was the most significant term enriched by the up-DEGs. For the GO terms at 40 days, ‘glucose import’ was the most significant term enriched by the up-DEGs. For the GO terms at 50 days, ‘abscisic acid-activated signaling pathway’ was the most significant term enriched by the up-DEGs (**Supplementary Table 6**). Interestingly, ‘protein-chromophore linkage’ and ‘photosynthesis, light harvesting in photosystem I’ were all significantly enriched at 25/30/40/50 days by down-DEGs. In total, we found that carbohydrate metabolism-, protein metabolism- and signal transduction-related processes were enriched among the up-DEGs, and photosynthesis-related processes were enriched among the down-DEGs. Above all, these results suggest that in oats growing under a long-day photoperiod, flowering and catabolic processes are induced, while vegetative growth is repressed.

### Identification of responses associated with flowering in oat under long-day and short-day conditions

**Figure 3:**
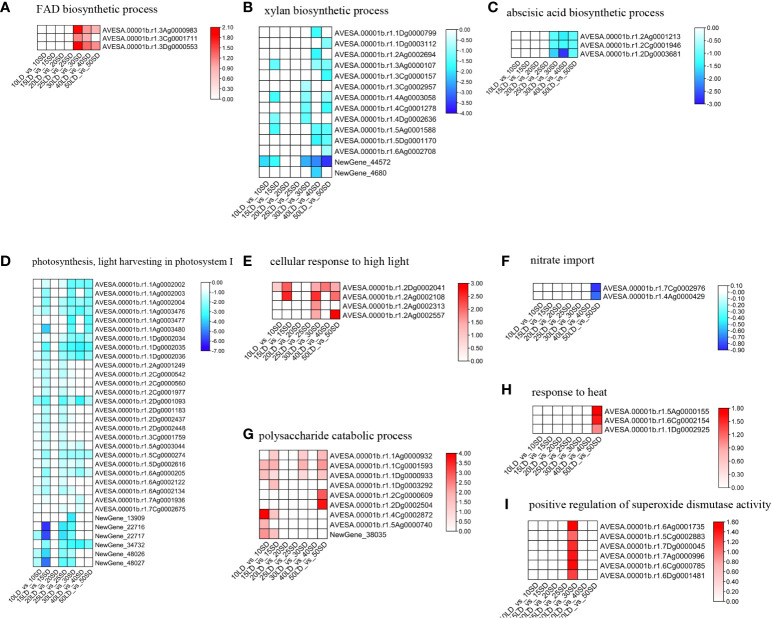
Expression levels of genes under ‘response to desiccation’ **(A)**, ‘xylan biosynthetic process’ **(B)**, ‘abscisic acid biosynthetic process’ **(C)**, ‘photosynthesis, light harvesting in photosystem I’ **(D)** ‘cellular response to high light intensity’ **(E)**, 'nitrate import' **(F)**, 'polysaccharide catabolic process' **(G)**, 'response to heat' **(H)**, and 'positive regulation of superoxide dismutase activity' **(I)** terms in oat lines under long-day and short-day photoperiods. The color scale indicates fold-change values (log2 values). The red and blue blocks illustrate the increased and decreased expression levels of genes under the long-day and short-day photoperiods, respectively.

To further investigate the flowering mechanism, we compared the expression patterns of genes under enriched terms in oat cultivar Baiyan 2 under different photoperiods. First, we compared the GO terms enriched from all different times. We found that there were several common terms shared by 30/40/50-day oat plants but not the same terms in the early flowering stage, indicating that oat could have a similar regulatory mechanism when in the late flowering stage (**Supplementary Figure 2**; **Supplementary Tables 6**, **7**). The ‘FAD biosynthetic process’ enriched by up-DEGs was shared in the 30/40/50 day GO analysis. First, the pathway related to the biosynthesis of FAD was examined. The BP term ‘FAD biosynthetic process’ (GO:0006747) included three of the same DEGs in the 30/40-day analysis, while the 50-day analysis included only two DEGs (**Figure 3A**; **Supplementary Table 8**). These three DEGs, whose products could be key enzymes, such as FAD synthesis in the FAD biosynthesis process, showed abundant expression in the long-day oat compared to the short-day oat. The upregulation of the FAD biosynthesis process was associated with flowering under a long-day photoperiod rather than under a short-day photoperiod in oats.

Furthermore, 12 terms enriched by the down-DEGs were shared in the 30/40/50-day GO analysis. For these terms enriched by the down-DEGs, the 12 BP terms could be classified into three groups. The first group was related to biosynthetic and metabolic processes, especially cell wall biogenesis-related processes, including the ‘xylan biosynthetic process’ and ‘cellulose biosynthetic process’. The second category was related to plant hormones, including ‘cellular response to jasmonic acid stimulus’ and ‘abscisic acid biosynthetic process’. The third was related to light response and photosynthesis, including ‘response to light stimulus’ and ‘photosynthesis, light harvesting in photosystem I’. The BP term ‘xylan biosynthetic process’ enriched 5, 7 and 9 down-DEGs in the 30-day, 40-day and 50-day analyses, respectively, suggesting that oat could decrease cell wall biogenesis (**Figure 3B**; **Supplementary Table 8**). Among them, 5 DEGs were shared by three analyses, which could be essential enzymes, such as xylan alpha-glucuronosyltransferase for xylan biosynthesis, showing decreased expression in long-day-photoperiod oats. For the ‘abscisic acid biosynthetic process’, there were three common DEGs that encoded zeaxanthin epoxidase and showed lower expression levels under the long-day photoperiod compared to the short-day photoperiod in the 30/40/50-day analysis (**Figure 3C**; **Supplementary Table 8**). Thirty-two, 14 and 13 downregulated DEGs were enriched in the 30-day, 40-day and 50-day analyses, respectively, showing increased expression in the short-day photoperiod under the ‘photosynthesis, light harvesting in photosystem I’ term (**Figure 3D**; **Supplementary Table 8**). All shared DEGs in these terms were related to the chlorophyll a-b binding protein family. The downregulation of cell wall biogenesis, hormone signals and light-related processes showed flowering induction in the long-day photoperiod oat compared with the short-day lines.

Moreover, in addition to the metabolism- and hormone-related processes, we also focused on light signal perception-, transduction- and photosynthesis-related processes, which are essential for flowering. The red light signal-related processes ‘red, far-red light phototransduction’ and ‘positive regulation of red or far-red light signaling pathway’ were enriched for the upregulated DEGs in the long-day photoperiod but not in the short-day photoperiod at 25 days and 30 days, respectively. The ‘detection of visible light’ was also enriched under the 25 days. Moreover, the BP term ‘cellular response to high light intensity’ was significantly enriched by upregulated DEGs in oat grown under the long-day photoperiod (**Figure 3E**; **Supplementary Table 8**). Interestingly, we found that ‘response to the light stimulus’ and ‘photosynthesis’ were enriched by the down-DEGs in all growth periods except 20 days. All genes mentioned before related to the chlorophyll a-b binding protein family in these terms were downregulated in the long-day period compared to the short-day period. Briefly, oats could enhance their light response and photosynthesis to maintain normal growth and development under a short-day photoperiod compared to a long-day photoperiod.

We know that flowering time could be associated with nutrient status, metabolic status and various chemicals in oat, so we examined several terms related to the above processes. Nitrogen regulation processes such as ‘nitrate import’ and ‘response to nitrate starvation’ were enriched for the down-DEGs under the short-day photoperiod but not under the long-day photoperiod. All two genes in these terms were upregulated in the short-day period compared to the long-day period, indicating that the oats grown under a long-day photoperiod showed lower nitrate concentrations and signal transduction than those grown under a short-day photoperiod (**Figure 3F**; **Supplementary Table 8**). The concentration of the soluble sugars and lipids could regulate the flowering time in oat. The upregulated terms ‘polysaccharide catabolic process’ and ‘glucan biosynthetic process’ were enriched in the long-day photoperiod oat but not in the short-day period. All five genes in these terms were upregulated in the long-day period compared to the short-day period (**Figure 3G**; **Supplementary Table 8**). Plants can flower earlier to escape various environmental stresses. Both heat and drought could accelerate flowering in oat. We also examined several terms related to abiotic and biotic stresses, such as ‘response to desiccation’, ‘response to heat’ and ‘response to fungus’. The oats grown under a long-day photoperiod showed higher expression of these terms than those grown under a short-day photoperiod (**Figure 3H** **).** In addition, we also found that the BP term ‘positive regulation of superoxide dismutase activity’ was enriched by the up-DEGs in the long-day photoperiod oat but not in the short-day photoperiod oat (**Figure 3I**; **Supplementary Table 8**). Superoxide dismutase could enhance flowering under a long-day photoperiod compared to a short-day photoperiod.

Taken together, these results suggest that the enrichment of sugar and nutrient biosynthesis- and metabolism-related processes, secondary metabolite-related processes and responses to external stress were associated with early flowering as well as the decrease in light-related processes and cell biogenesis.

### The responses of plant hormones to flowering in oat under different photoperiods

Plant hormones are critical for growth and development throughout plant life. It could determine the flowering time by regulating the concentration, the signal and the release position. Plants have different flowering times under different environmental factors according to the crosstalk of various plant hormones. Here, we examined the plant hormone-related BP terms under different photoperiods to determine the feasible regulatory mechanisms.

**Figure 4:**
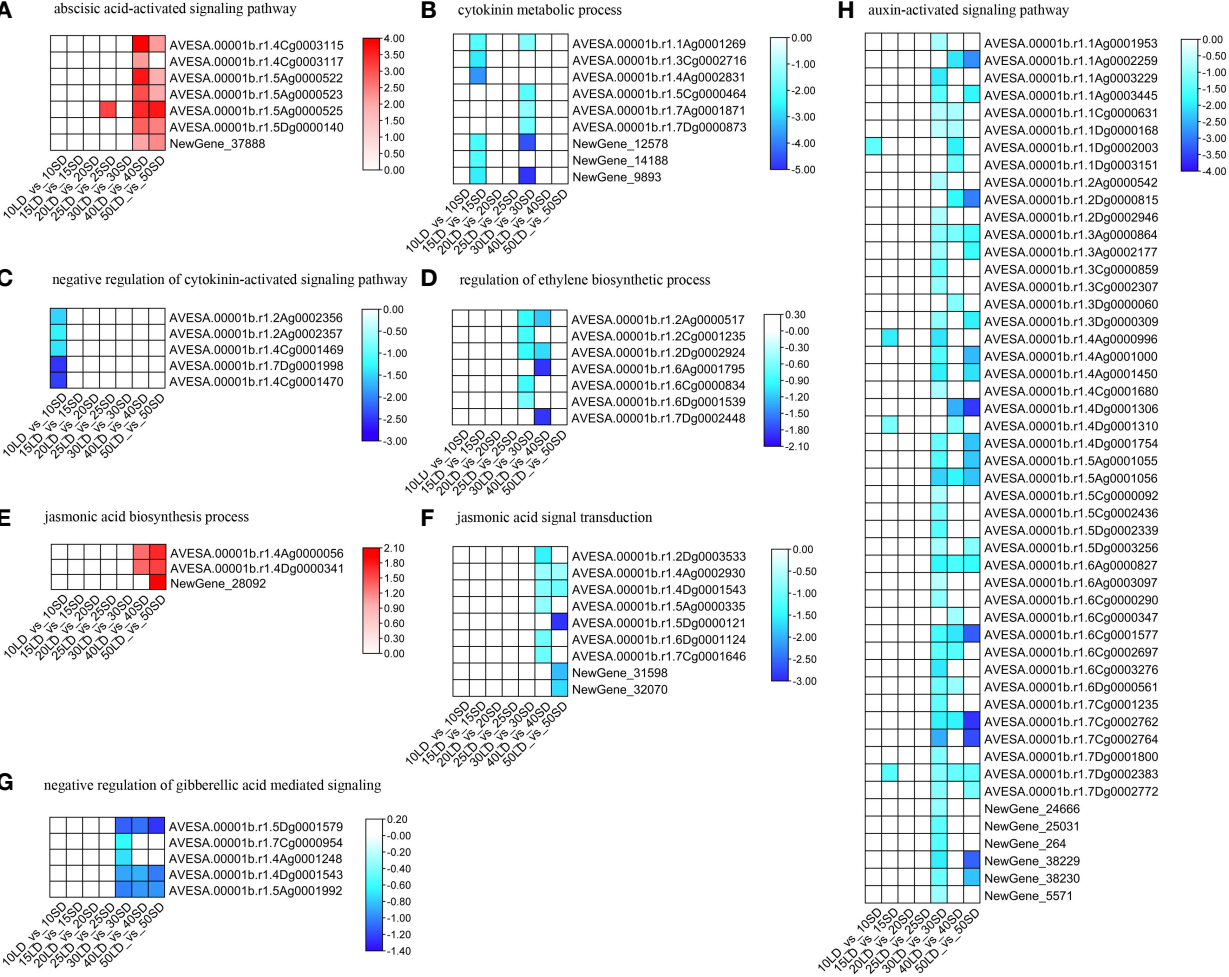
Expression levels related to plant hormones, including ABA **(A)**, CK **(B, C)**, ET **(D)**, JA **(E, F)**, GA **(G)**, and auxin **(H)**, in oat cultivars under long-day and short-day photoperiods. The annotations of the color scale and blocks were the same as in **Figure 3**.

ABA-related terms were not induced before 30 days except at 15 days, suggesting that ABA had no effect on flowering or was balanced in plants in the early stage. The BP term ‘abscisic acid biosynthetic process’ was enriched by the down-DEGs under the 30/40/50-day treatments (**Figure 3C**; **Supplementary Table 8**), while the term ‘abscisic acid-activated signaling pathway’ was enriched by the up-DEGs under 40/50 days (**Figure 4A**; **Supplementary Table 8**). All these data indicated that the downregulation of ABA biosynthesis and upregulation of ABA signal transduction could accelerate flowering in oat under a long-day photoperiod.

None of the cytokinin-related BP terms were enriched by the up-DEGs in all growth periods, suggesting that oat does not need higher cytokinin concentrations for flowering. However, the ‘cytokinin metabolic process’ and ‘negative regulation of cytokinin-activated signaling pathway’ were enriched by the down-DEGs (**Figures 4B, C**; **Supplementary Table 8**). Cytokinin biosynthesis and signal transduction were not upregulated, but metabolism-related processes were downregulated, indicating that oats need the proper cytokinin concentration for early flowering under a long-day photoperiod as well as to maintain growth and development.

Ethylene showed similar responses to those of cytokinin. The ‘regulation of ethylene biosynthetic process’ was enriched by the down-DEGs (**Figure 4D**; **Supplementary Table 8**), suggesting that oats could decrease the internal content of ethylene to induce flowering under long-day photoperiod conditions rather than under short-day conditions.

Generally, jasmonic acid biosynthesis-related processes were enriched by upregulated DEGs (**Figure 4E**; **Supplementary Table 8**), while jasmonic acid signal transduction was enriched by downregulated DEGs (**Figure 4F**; **Supplementary Table 8**). Oats increase jasmonic acid concentrations for flowering under a long-day photoperiod. The higher jasmonic acid concentration could repress other hormones for flowering. Meanwhile, the downregulation of the JA signaling process could also enhance the above process.

In addition, we found that the BP term ‘negative regulation of gibberellic acid mediated signaling pathway’ was enriched by the down-DEGs under a short-day photoperiod (**Figure 4G**; **Supplementary Table 8**), indicating that gibberellin could enhance the early flowering process.

Similar to cytokinin, there were no enriched terms related to auxin for the up-DEGs. The BP term ‘auxin-activated signaling pathway’ was only enriched in the down-DEGs at 30/40/50 days (**Figure 4H**; **Supplementary Table 8**), indicating that oat should have stable auxin homeostasis for growth and development. In addition, the downregulation of auxin signal transduction could be associated with flowering. There could be one or two other hormones that could be downregulated for auxin signal transduction, thus leading to flowering under a long-day photoperiod.

Finally, it is a complex and coordinating plant hormone response mechanism to regulate the flowering time in oat under long-day and short-day photoperiods. Several hormones, such as cytokinin, regulate growth and development at the early stage, while ABA and auxin show enhanced functions in flowering time at the late stage.

To confirm the accuracy of the RNA-Seq results, we analyzed the expression levels of ten key genes involved in the six hormone-related pathways by qRT-PCR for leaf samples collected at different time points. The relative gene expression of these genes showed a similar trend as that of the FPKM values at different time points (**Supplementary Figure 3**), indicating that they were in accordance with the RNA-Seq results.

### KEGG enrichment analysis of differentially expressed genes

In addition to GO analysis, KEGG pathway analysis was also performed to investigate the biological pathways involved in these DEGs. A total of 4 (1 for up-DEGs and 3 for down-DEGs), 16 (8 for up-DEGs and 8 for down-DEGs), 2 (2 for up-DEGs), 16 (12 for up-DEGs and 4 for down-DEGs), 48 (28 for up-DEGs and 20 for down-DEGs), 25 (15 for up-DEGs and 10 for down-DEGs), and 23 (15 for up-DEGs and 8 for down-DEGs) KEGG pathways were significantly enriched after growth in a long-day photoperiod for 10/15/20/25/30/40/50 days in oats (**Supplementary Table 9**).

**Figure 5:**
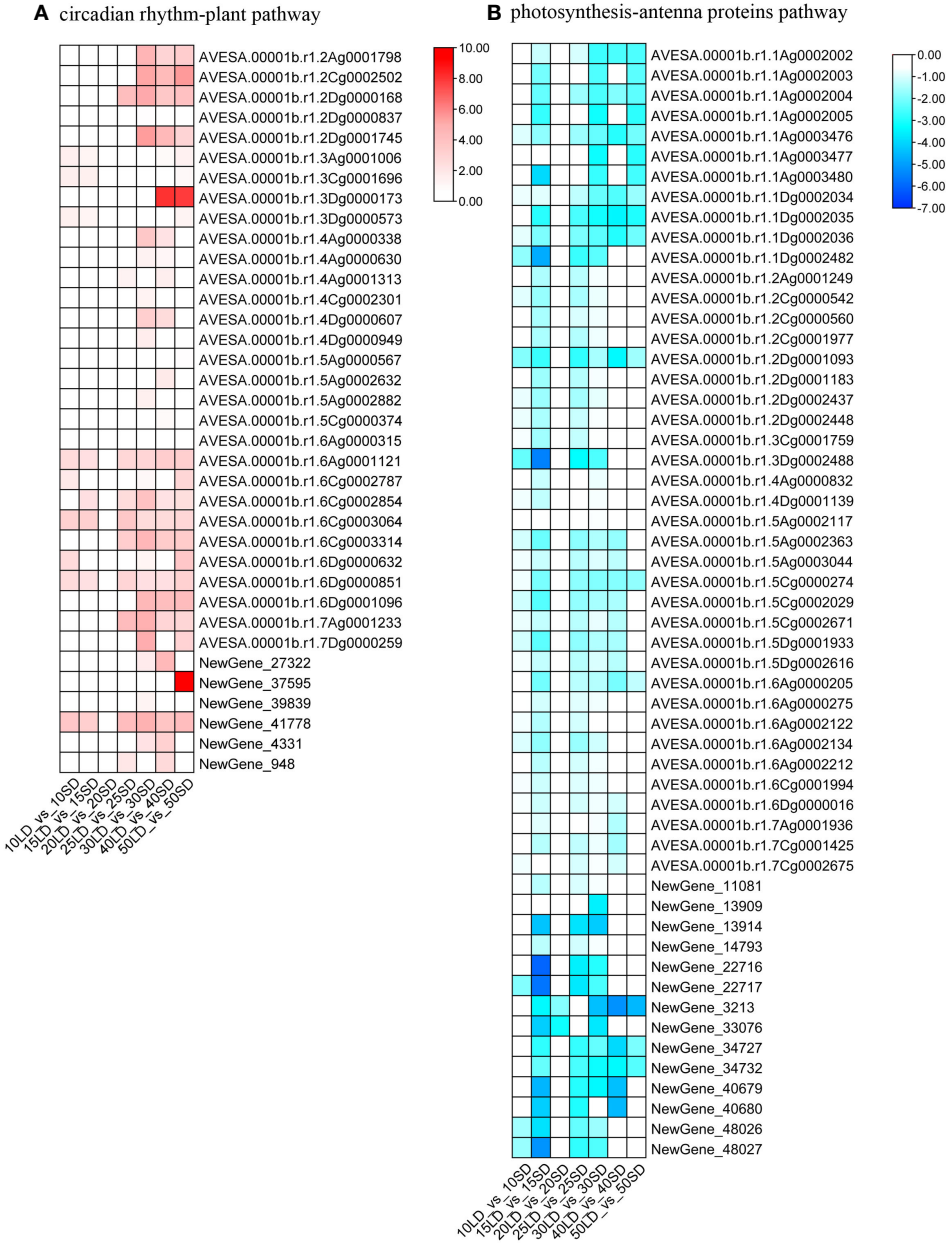
Heatmaps illustrating the expression levels of genes involved in the circadian rhythm-plant pathway **(A)** and photosynthesis-antenna proteins pathway **(B)** under long-day and short-day photoperiods. The annotations of the color scale and blocks were the same as in **Figure 3**.

By comparing the KEGG pathways of the seven groups, we found no common pathways among them (**Supplementary Figure 4**). Then, we compared the KEGG pathways enriched from 30/40/50 days of photoperiod induction. There were five pathways enriched by up-DEGs shared in the 30/40/50-day analysis (**Supplementary Figure 4A**). Among them, four pathways are related to substance metabolism, including ko00330 (arginine and proline metabolism), ko00280 (valine, leucine and isoleucine degradation), ko00410 (beta-alanine metabolism) and ko00903 (limonene and pinene degradation), indicating that the metabolic processes of amino acids and terpenes were very active in the flowering process, and these activities may provide more nutrients or signaling substances for oat flowering (Chen et al., 2016; Gawarecka and Ahn, 2021). Moreover, there was one shared pathway, ‘Circadian rhythm-plant’, in the 30/40/50-day KEGG analysis, which had been verified to be related to plant flowering time in previous studies. Twenty-six, 23 and 21 upregulated DEGs were enriched in the 30/40/50-day photoperiod inducement, respectively (**Figure 5A**; **Supplementary Table 8**). The results suggested that these circadian clock-related genes are induced by a long-day photoperiod, and their increased expression is associated with early flowering.

In addition, we found only one KEGG pathway, ‘Photosynthesis-antenna proteins’, enriched by down-DEGs shared in the 30/40/50-day analysis (**Supplementary Figure 4B**). A total of 54, 24 and 16 downregulated DEGs were enriched in the 30-day, 40-day and 50-day photoperiod treatments, respectively, indicating that the decreased expression levels of photosynthesis-related oat genes were related to the early flowering of oat (**Figure 5B**; **Supplementary Table 8**). This result was consistent with the results of GO analysis.

### Identification of transcription factors in response to long-day photoperiod conditions

Transcription factors play crucial roles in the control of photoperiod-dependent flowering. Therefore, we submitted the DEGs from the seven comparison groups to the iTAK database to predict the transcription factors. A total of 701 TFs were identified and assigned to 44 different families (**Supplemental Table S10**). The identified TF families with the top 10 numbers of DEGs included MYB (62), AP2/ERF (59), bHLH (57), C2C2 (51), HB (48), WRKY (47), NAC (47), B3 (30), bZIP (30), and AUX/IAA (29) (**Supplementary Table 10**). Previous studies have reported that several transcription factors can not only specifically regulate flowering time through various pathways, such as the photoperiodic pathway and hormonal pathways but also via crosstalk with these pathways.

### MapMan metabolic pathway analysis of DEGs

To further understand the details of the oat flowering mechanisms that are affected by the long-day photoperiod, the DEGs of seven comparison groups were mapped into metabolic pathways by using MapMan.

**Figure 6:**
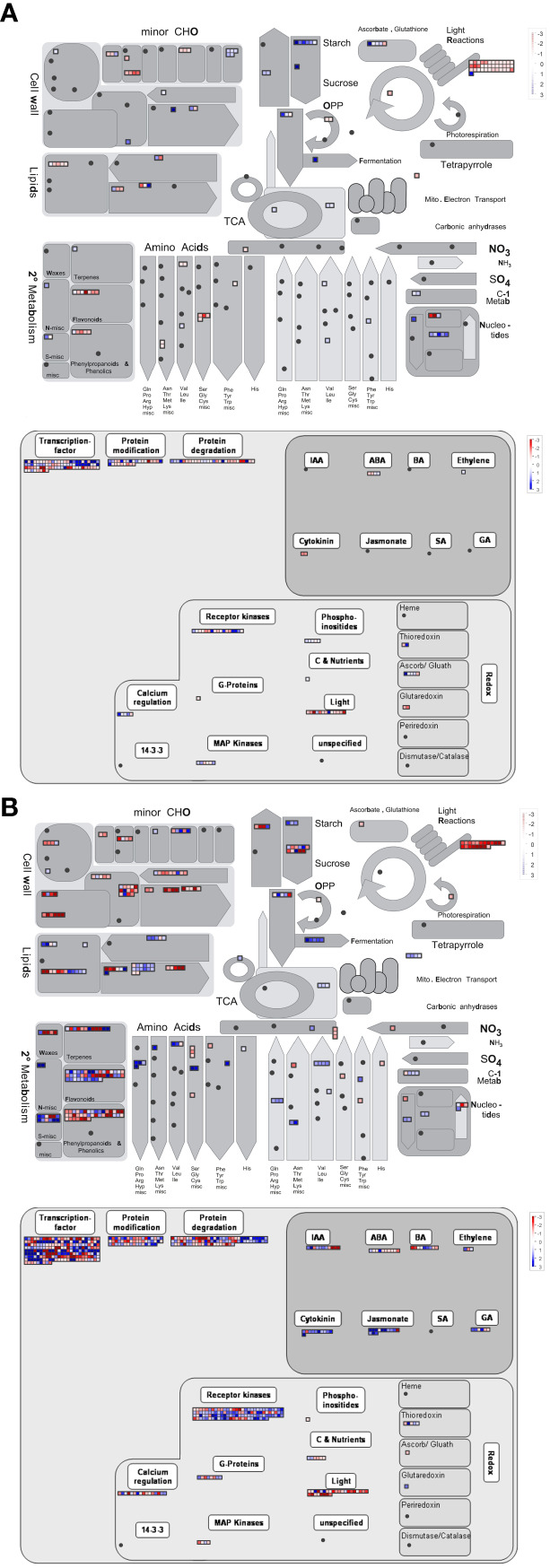
MapMan annotation showing the metabolic and regulatory pathways after 10 days **(A)** and 50 days **(B)** of photoperiod induction. Each square represents a differentially expressed gene. The blue and red lattices represent upregulated and downregulated genes, respectively. The color scale represents the log_2_fold change.

Given the significant changes in metabolism-related DEGs and pathways after long-day photoperiod treatments, metabolic and regulatory maps were constructed to display an overview of the DEGs of the seven comparison groups (**Figure 6**; **Supplementary Figure 5**). We found that many metabolic and biological pathways were very active after long-day photoperiod treatments, especially photosynthesis and related metabolisms, major and minor CHO metabolisms, secondary metabolism, and plant hormone metabolism. In addition, the number of photoperiod-responsive genes was greatly increased after long-day photoperiod induction.

In our study, 53 genes involved in the sucrose metabolism process, including six sucrose phosphate synthase genes, thirty-one sucrose invertase genes, five sucrose hexokinase genes and eight sucrose fructokinase genes, were significantly changed under long-day photoperiodic induction of oat (**Supplementary Figure 6**; **Supplementary Table 11**). Two and one sucrose phosphate synthase (SPS) genes were upregulated under 10LD_vs_10SD and 20LD_vs_20SD, respectively. Under 30LD_vs_30SD, there was one upregulated gene and two downregulated SPS genes. There were three downregulated SPS genes under 40LD_vs_40SD. In addition, under 10LD_vs_10SD and 15LD_vs_15SD, the expression of most genes encoding sucrose invertase was upregulated, and more genes were downregulated after 30 days of photoperiod induction. Moreover, five genes encoding sucrose hexokinase and eight genes encoding sucrose fructokinase showed similar expression trends as the sucrose invertase genes. These results indicate that there was a very active sucrose metabolism process during oat flowering, and this process is closely related to the flowering of oat.

## Discussion

**Figure 7:**
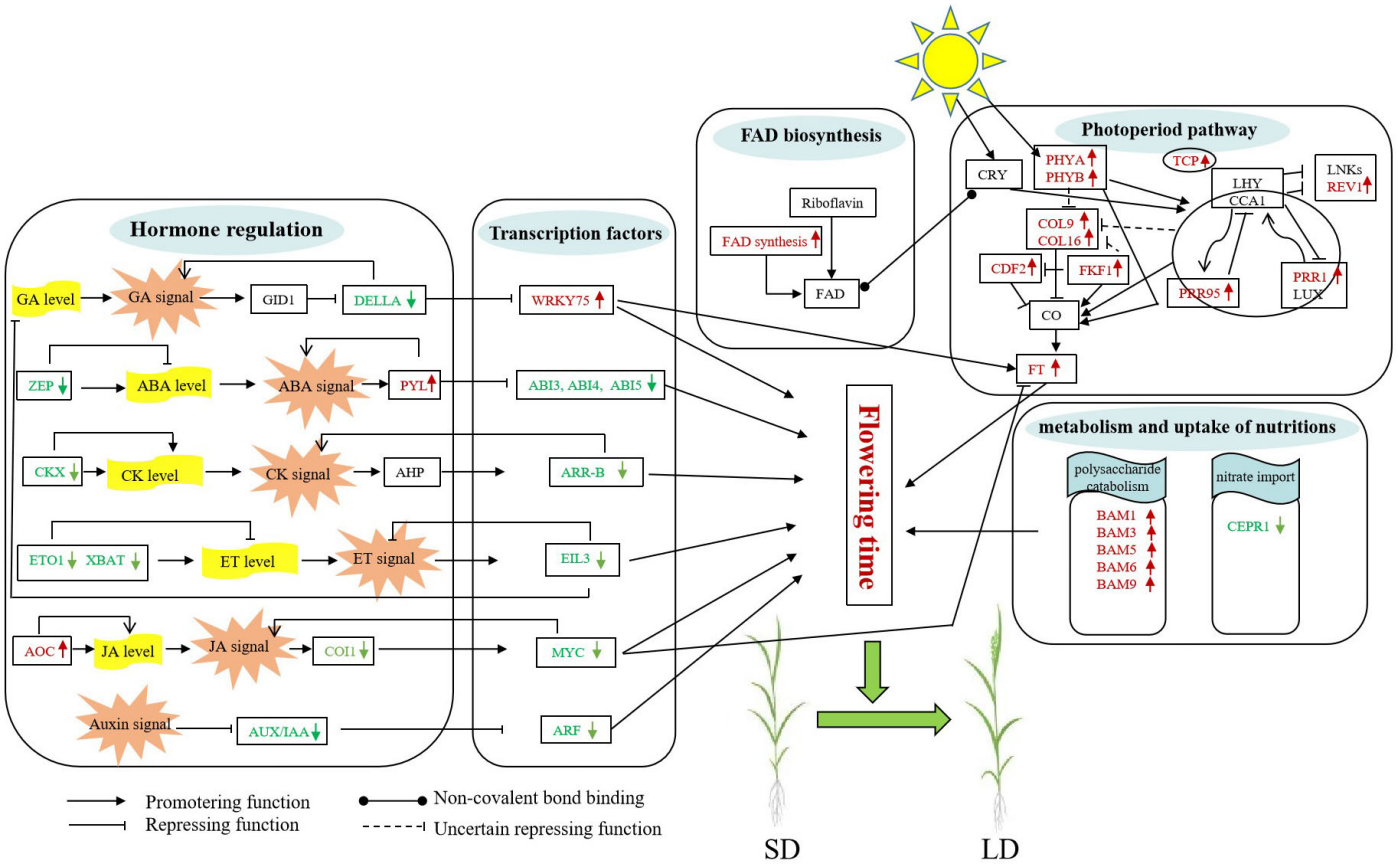
Possible flowering molecular model of oat under long-day photoperiod inducement. The green and red fonts represent downregulated and upregulated genes, respectively. The full names of all genes noted in this model are listed in **Supplementary Table 12**.

Flowering is a complex trait that determines crop performance under field conditions. Photoperiod is one of the major cues that affect flowering behavior in temperate cereals (Kurokura et al., 2013; Osnato et al., 2022). However, no efforts have been made toward a comprehensive understanding of the molecular mechanism of photoperiodic flowering in less studied species such as oat. In this study, we performed transcriptomic analyses on the photoperiod-sensitive variety Baiyan 2 at different growth stages to detect the mechanism of flowering under different photoperiods. Combined with previous studies, we proposed a hypothetical model for the flowering induction response of photoperiod-sensitive oat cultivars under long-day induction (**Figure 7**). Flowering for a long-day variety of oat under long-day conditions is regulated by multiple exogenous and endogenous signals including circadian clock, photoperiod, plant hormones, sugar metabolism, and others.

### The hormone pathways and the core transcription factors involved in these pathways collaboratively regulate oat flowering

Various hormones have been reported to be related to the complex regulation of flowering time in plants (Ionescu et al., 2017; Izawa, 2021). Based on the Gene Ontology enrichement analysis, the current results showed that the flowering transition requires alterations in hormone metabolism and signaling, including GA, ABA, auxin, CK, JA and ethylene.

The gibberellin pathway is one of the main mechanisms of plant flowering control. The obstruction of the GA signal transduction pathway will directly affect the flowering of plants (Bao et al., 2020). The growth inhibitors DELLAs are the main component of GA signaling, and they can interact with WRKY transcription factors to inhibit WRKY activation, such as WRKY75, finally affecting flowering by promoting FT expression in leaves (Li et al., 2016; Gao et al., 2017; Zhang et al., 2018). In our study, the expression of DELLA genes was downregulated under the 30/40/50-day treatments, suggesting that GA signals could be sensed faster under long-day conditions than under short-day conditions, activating the expression of downstream genes, including WRKY75, finally leading to early flowering in oats under long-day conditions.

Although ABA is often thought to be a stress-related hormone, it also plays an important role in plant development (Xiong and Zhu, 2003; Sah et al., 2016). Both positive and negative regulation of flowering time by ABA have been reported (Domagalska et al., 2010; Conti et al., 2014). Previous studies have shown that ABA treatment greatly delays flowering (Wang et al., 2013). In our study, long days resulted in significant changes in the expression levels of ABA-related genes. The Zeaxanthin epoxidase (ZFP) gene, which is involved in the first step of ABA biosynthesis, was downregulated. Moreover, the ABA receptor PYRABACTIN RESISTANCE 1-LIKE (PYL) was upregulated during the flowering process, indicating that the ABA signaling pathway was related to the long-day flowering of oat. Subsequently, the ABA hormone induces the expression of bZIP transcription factors, such as ABI4 and ABI5, in the ABA transduction pathway. Previous studies have shown that ABI5 and ABI4 repress flowering by directly promoting the expression of FLC, which in turn results in reduced expression of FT and SOC1 (Lopez-Molina et al., 2003; Bossi et al., 2009; Shu et al., 2016; Shu et al., 2018). Our results showed that the expression levels of ABI4 and ABI5 decreased under a long-day photoperiod, therefore inducing oat flowering.

Cytokinin plays an important role in the induction of flower formation and the regulation of flowering time (Kieber and Schaller, 2014). In *A. thaliana*, the overexpression lines of the cytokinin oxidase/dehydrogenase gene reduce endogenous CK content, and exhibit late-flowering phenotypes under long-day conditions and no-flowering phenotypes under short-day conditions (Bartrina et al., 2017). In the current study, the cytokinin dehydrogenase genes (CKXs), key genes of CK degradation, were downregulated during long days, therefore leading to the relatively low cytokinin content of oat during short days and the failure to flower, while the normal accumulation of cytokinin in long days led to the flowering of oat. In addition, the downregulation of type-B ARABIDOPSIS RESPONSE REGULATOR (ARR-B) factors, which negatively regulate the cytokinin signal transduction pathway, resulted in a strong response to cytokinin in oat and triggered flowering under a long-day photoperiod.

JA regulates multiple plant growth responses, such as flowering-related processes (Wasternack and Hause, 2013; Griffiths, 2020; Zhao et al., 2022). In the current study, the genes involved in the JA biosynthesis and signaling pathways were upregulated and downregulated, respectively. The F-box protein CORONATINE INSENSITIVE 1 (COI1) mediates the degradation of JASMONATE‐ZIM domain (JAZ) proteins to release JAZ-bound TFs, such as MYC2 (Zhai et al., 2015; Browse and Wallis, 2019). These MYCs could repress the transcription of the *FT* gene by genetically interacting with this gene, therefore delaying flowering time (Cheng et al., 2011; Wang et al., 2017). In this study, the COI1 genes and MYC2 genes were downregulated, indicating that the inhibition of flowering by jasmonic acid decreased under long days, leading to flowering of oat.

Previous research has shown that increased ethylene content in plants causes delayed flowering under both long and short days (Achard et al., 2006; Achard et al., 2007). In the current study, downregulated expression of genes associated with ethylene synthesis resulted in less ethylene accumulation under long days than under short days, which may be the reason why oats flower under long days, supporting a previous report that ethylene is a flowering inhibitor (Bao et al., 2020). In addition, in the ethylene signaling pathway, the center regulator EIN3-like (EIL) transcription factors were downregulated, indicating that negative regulation of the ethylene signal transduction pathway resulted in flowering under long days, which was in line with the negative role of ethylene.

Auxin is the earliest identified plant hormone, which affects many physiological processes such as the elongation and differentiation of plant cells and the growth and development of roots and leaves, and participates in the regulation of flower formation (Kasahara, 2016). In the present study, the expression of auxin signal transduction pathway genes, including AUX/IAA and auxin response factor (ARF) transcription factors, decreased under long days, suggesting that oat plants were less sensitive to the auxin response under long days, promoting oat flowering. In summary, our results revealed that multiple phytohormones play important roles specifically and synergistically in the long-day photoperiod inducement of flowering in oat.

### Sugar metabolism and nutrition import were the necessary processes for oat flowering under the induced photoperiod

A long-day photoperiod affects the metabolic process of nutrients in the leaves (Lauxmann et al., 2016). Carbohydrates are the final product of photosynthesis and an important energy source in the plant life cycle. In addition, as important signaling molecules, they help plant species adapt to changes in the surrounding environment and coordinate their growth and development (Cho et al., 2018; Wingler, 2018). In particular, sucrose, as the main source of energy, participates in plants from the vegetative stage to the reproductive stage (Yoon et al., 2020).

Through MapMan functional analysis, we found that the expression levels of several genes encoding sucrose phosphate synthase, invertase, hexokinase and fructokinase were significantly changed. At the early flowering stage, the sucrose phosphate synthase, sucrose invertase genes and hexokinase genes were upregulated, providing a large amount of energy reserve for plants, therefore promoting early flowering of plants. When entering the late flowering period, the expression of all these genes gradually decreased, indicating that the plant may need only a small amount of carbohydrates to ensure flowering and that some sucrose may accumulate in this period. Moreover, sucrose can also play a regulatory role in the flowering process of plants as a signal molecule, which may also be one of the reasons why oats flower under long days (Gawarecka and Ahn, 2021). In addition, starch metabolism and biosynthetic processes are involved in the plant flowering pathway. In our study, β-amylase (BAM) genes involved in starch degradation were upregulated in oat through GO analysis. These genes participate in the metabolic process of carbohydrates, increasing the content of soluble sugars in leaves and therefore providing nutrients and energy for plant growth, development and flowering. Additionally, nitrogen sources are thought to play a crucial role in plant developmental processes, including the regulation of flowering time (Zhang et al., 2021; Zhang et al., 2023). Our study showed that the CEPRECEPTOR1 (CEPR1) gene, which is involved in nitrate absorption and signal transduction (Ota et al., 2020; Taleski et al., 2020), was downregulated and reduced nitrogen source substances in oat, resulting in early flowering under a long-day photoperiod. These results suggested that the changes in these genes may induce sugar and nitrogen signals by modulating nutrition metabolism processes, therefore regulating the flowering time of Baiyan 2 under a long-day photoperiod.

### The circadian clock system regulates the flowering process of oat

The circadian clock system can coordinate external light, temperature signals and internal metabolic developmental signals to output circadian rhythm signals, affecting almost all plant growth, development and metabolism processes (Inoue et al., 2018; Creux and Harmer, 2019). Therefore, this system was often separated into three parts, including the input pathway, central oscillator and output pathway. Among the downstream responses of clock-regulated output pathways, the photoperiodic flowering response is the most representative.

FAD is a light-harvesting chromophore of cryptochrome that can noncovalently bind with cryptochromes (CRYs) and regulate the flowering process of plants through the photoperiodic pathway (Bouly et al., 2007; Palayam et al., 2021). In our study, long days induced the upregulated expression of the FAD synthetase gene, and the increased expression level of this gene may affect the input pathway, therefore promoting oat flowering under a long-day photoperiod. In addition, long days also affected a series of flowering-related genes, including several light photoreceptor phytochromes (PHYs) (Song et al., 2018; Hu et al., 2020), PSEUDO-RESPONSE REGULATOR 95 (PRR95), REV1 (REVEILLE 1), and PSEUDO-RESPONSE REGULATOR 1 (PRR1) involved in circadian rhythms (Shim et al., 2016), CDF2, FKF1, and CONSTANS-LIKEs (COLs) which are the central regulators. These genes were rapidly induced during the flowering process, therefore activating a large amount of FT gene expression in the leaves, which in turn promotes flowering.

## Data availability statement

The datasets presented in this study can be found in online repositories. The names of the repository/repositories and accession number(s) can be found below: The raw data was uploaded to the NCBI Sequence Read Archive (http://www.ncbi.nlm.nih.gov/) with the accession number PRJNA997076.

## Author contributions

MZ: Conceptualization, Formal Analysis, Investigation, Writing – original draft, Writing – review & editing. YJ: Conceptualization, Writing – review & editing, Formal Analysis, Writing – original draft. HD: Conceptualization, Writing – review & editing. XS: Writing – review & editing, Data curation. JT: Writing – review & editing. MS: Writing – review & editing. FM: Writing – review & editing. CR: Writing – review & editing, Conceptualization, Project administration, Supervision. YY: Writing – review & editing, Conceptualization, Project administration, Supervision.

## Supplementary material

Supplementary Figure 1 Photograph of the oat inflorescence at the heading date stage. Black bar=5 cm.

Supplementary Figure 2 Venn diagram showing the shared enriched BP terms for the up-DEGs **(A)** and the down-DEGs at different time points. The significant GO terms with FDR ≤ 0.05. 10&15d represents 10LD_vs_10SD and 15LD_vs_15SD, 20d, 25d, 30d, 40d and 50d represents 20LD_vs_20SD, 25LD_vs_25SD, 30LD_vs_30SD, 40LD_vs_40SD and 50LD_vs_50SD, respectively.

Supplementary Figure 3 Comparison between RNA-Seq results and qRT-PCR validation of hormone-related genes. * and ** represent significant differences at 0.01<P<0.05 and P ≤ 0.01, respectively. The P value was calculated using IBM SPSS statistics 19 software. The bar height represents the mean values, and error bars indicate standard deviation.

Supplementary Figure 4 Venn diagram showing the shared enriched KEGG pathways for the up-DEGs **(A)** and the down-DEGs **(B)** at different time points. The significant KEGG pathways with FDR ≤ 0.05. The annotations at 10&15d, 20d, 25d, 30d, 40d and 50d were the same as those in **Supplementary Figure 2**.

Supplementary Figure 5 Overview of the metabolic and regulatory pathways of differentially expressed genes after 15 days **(A)**, 20 days **(B)**, 25 days **(C)**, 30 days **(D)**, and 40 days **(E)** of photoperiod induction. Each square represents a differentially expressed gene. The blue and red lattices represent upregulated and downregulated genes, respectively. The color scale represents the log2fold change.

Supplementary Figure 6 The heat-map expression profiles of the differentially expressed genes involved in sucrose metabolism. The red and blue blocks illustrate the increased and decreased expression levels of genes under the long-day and short-day photoperiods, respectively.
