# Supplementary figures and images for: Transcriptomic response for revealing the molecular mechanism of oat flowering under different photoperiods
Source: Front Plant Sci. 2023 Oct 31;14:1279107. doi: 10.3389/fpls.2023.1279107 (PMC10644674; doi:10.3389/fpls.2023.1279107)

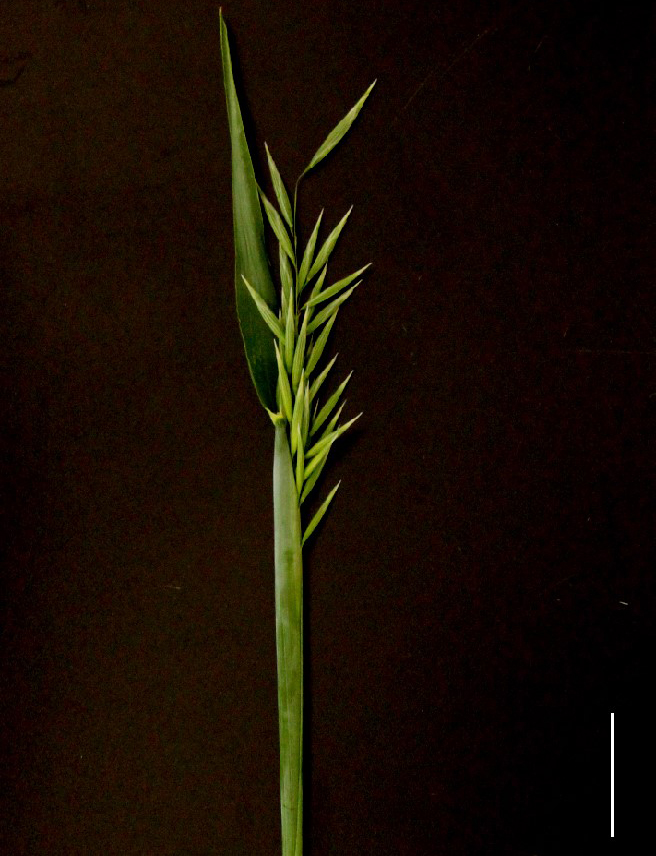

Supplement: Supplementary Figure 1 — Photograph of the oat inflorescence at the heading date stage. Black bar=5 cm. [file Image_1.jpeg]

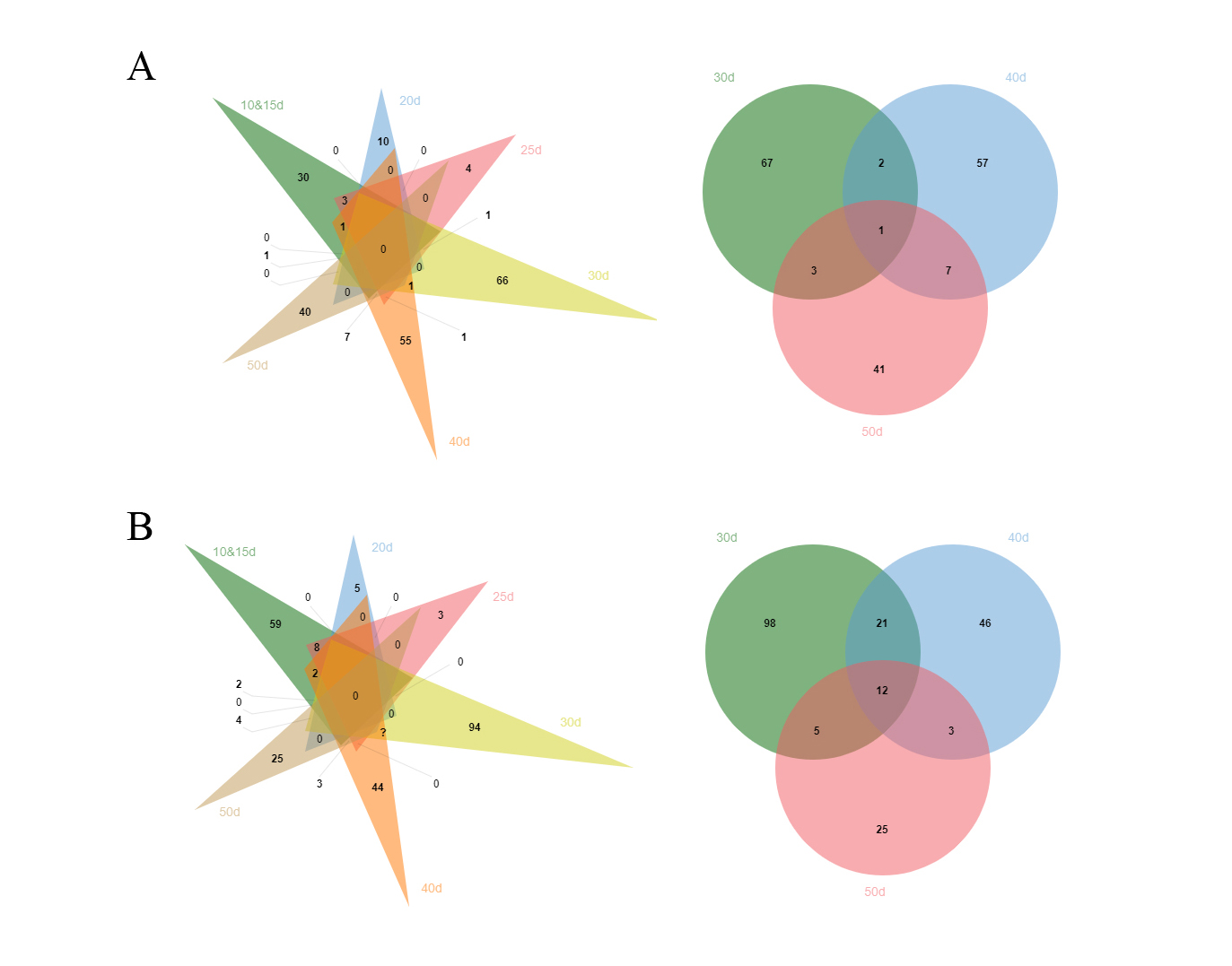

Supplement: Supplementary Figure 2 — Venn diagram showing the shared enriched BP terms for the up-DEGs (A) and the down-DEGs at different time points. The significant GO terms with FDR ≤ 0.05. 10&15d represents 10LD_vs_10SD and 15LD_vs_15SD, 20d, 25d, 30d, 40d and 50d represents 20LD_vs_20SD, 25LD_vs_25SD, 30LD_vs_30SD, 40LD_vs_40SD and 50LD_vs_50SD, respectively. [file Image_2.jpeg]

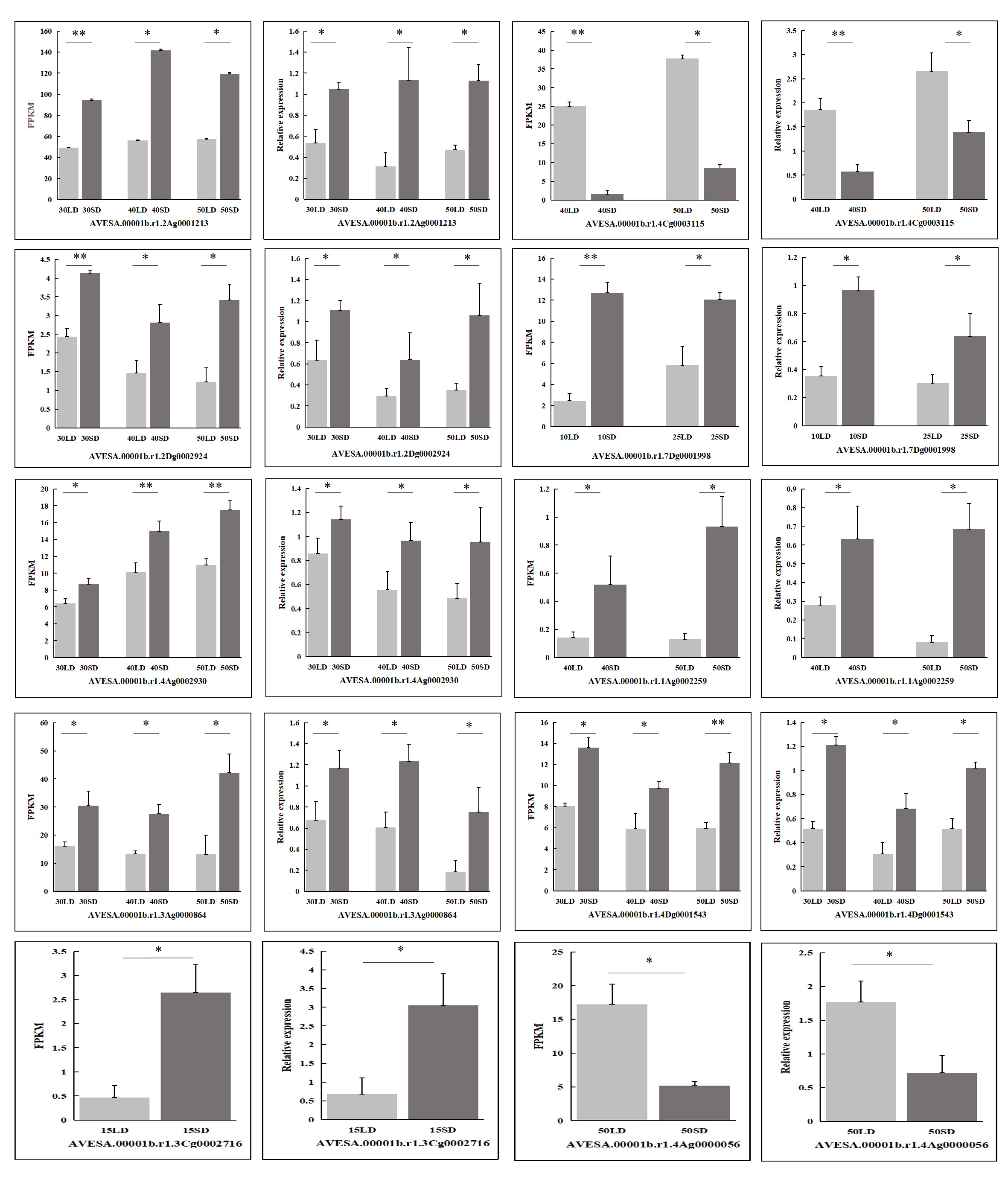

Supplement: Supplementary Figure 3 — Comparison between RNA-Seq results and qRT-PCR validation of hormone-related genes. * and ** represent significant differences at 0.01<P<0.05 and P ≤ 0.01, respectively. The P value was calculated using IBM SPSS statistics 19 software. The bar height represents the mean values, and error bars indicate standard deviation. [file Image_3.jpeg]

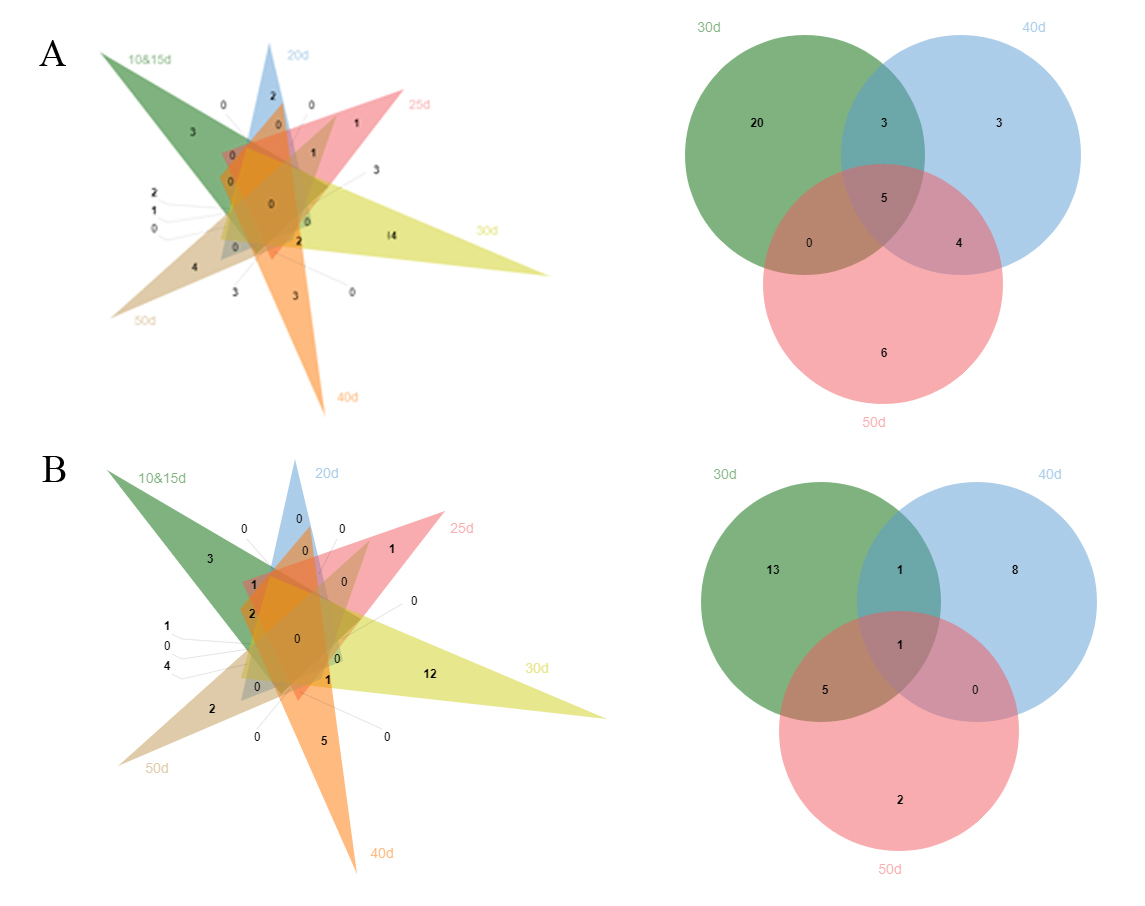

Supplement: Supplementary Figure 4 — Venn diagram showing the shared enriched KEGG pathways for the up-DEGs (A) and the down-DEGs (B) at different time points. The significant KEGG pathways with FDR ≤ 0.05. The annotations at 10&15d, 20d, 25d, 30d, 40d and 50d were the same as those in Supplementary Figure 2 . [file Image_4.jpeg]

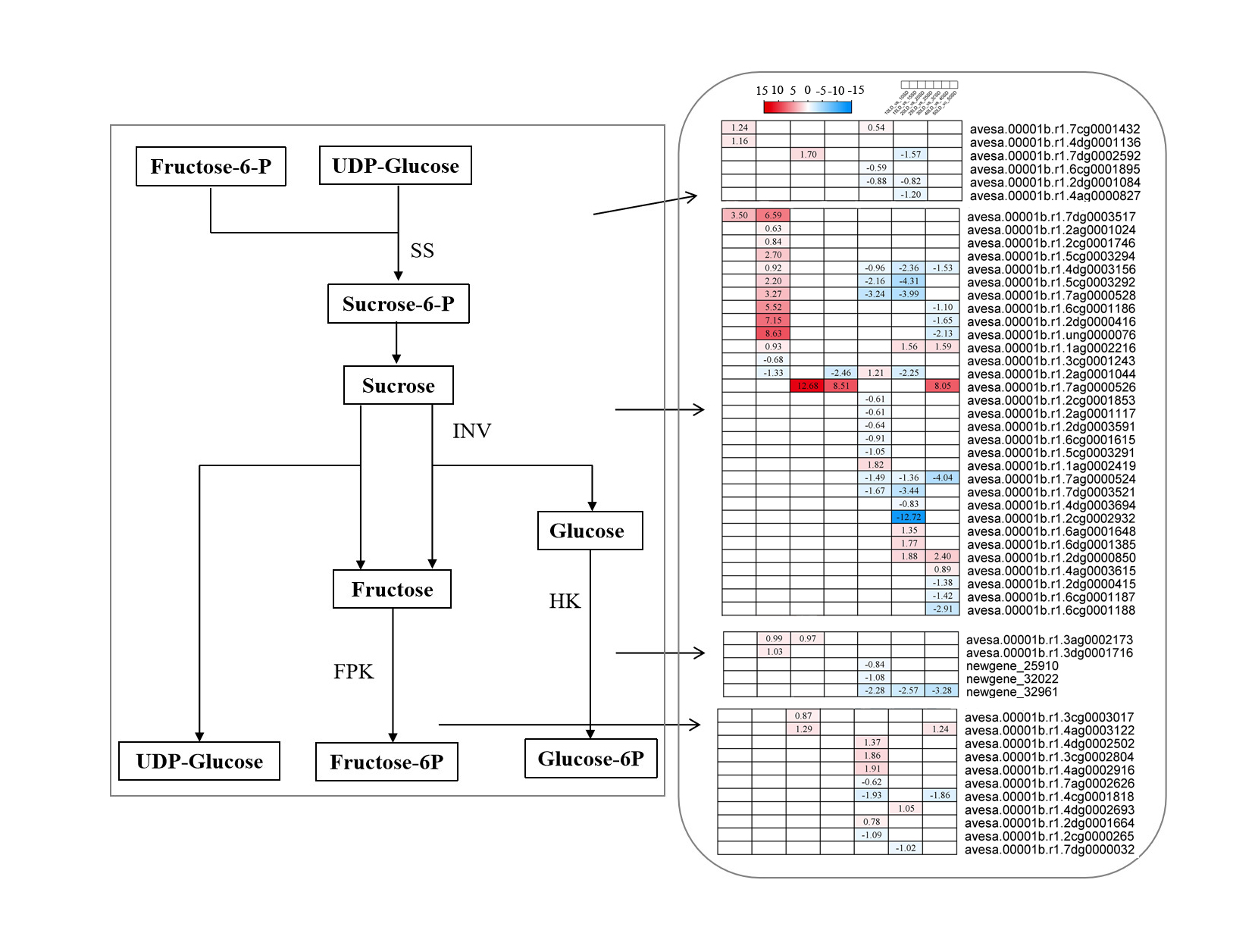

Supplement: Supplementary Figure 6 — The heat-map expression profiles of the differentially expressed genes involved in sucrose metabolism. The red and blue blocks illustrate the increased and decreased expression levels of genes under the long-day and short-day photoperiods, respectively. [file Image_6.jpeg]
